# Compassion fatigue and stress related to cardiopulmonary resuscitation: a study of critical care nurses’ experiences

**DOI:** 10.1186/s12912-023-01640-y

**Published:** 2023-12-18

**Authors:** Ayman Mohamed El-Ashry, Shimmaa Mohamed Elsayed, Mohamed Adel Ghoneam, Mohamed Hussein Ramadan Atta

**Affiliations:** 1 Psychiatric and Mental Health Nursing Department, Faculty of Nursing, Alexandria University, Alexandria, Egypt; 2 Critical Care and Emergency Nursing Department, Faculty of Nursing, Damanhour University, Damanhour, Egypt; 3 Critical Care and Emergency Nursing Department, Faculty of Nursing, Beni-Suef University, Beni-Suef, Egypt

**Keywords:** Critical care nurses, Compassion fatigue, Post-code stress, CPR, Hospital

## Abstract

**Background:**

Cardiopulmonary resuscitation (CPR) is considered one of the most stressful experiences in critical care nursing; it directly and indirectly leads to compassion fatigue and burnout.

**Aim:**

Determine the levels of and relationship between postcode stress and compassion fatigue.

**Design and methods:**

A descriptive-correlational study using 300 critical care nurses from five intensive care units in two hospitals was conducted.

**Tools:**

Demographic and work-related data, the Postcode Stress Scale, and the Professional Quality of Life Scale: Compassion Fatigue subscale.

**Results:**

Nurses had moderate to high postcode stress and compassion fatigue (67.98 ± 16.39 and 65.40±14.34, respectively). Moreover, there was a significant positive correlation between postcode stress, burnout (*r*=0.350, *p*=<0.001), secondary traumatic stress (*r*=0.518, *p*=<0.001), and subsequently, compassion fatigue (*r*=0.449, *p*=<0.001). In addition, higher levels of postcode stress were associated with higher levels of compassion fatigue with its subscales: burnout and secondary traumatic stress, with a coefficient of determination for compassion fatigue (0.199), burnout subscale (0.121), and secondary traumatic stress (0.266).

**Conclusion:**

Critical care nurses involved in resuscitation experiences are susceptible to postcode stress, burnout, secondary traumatic stress, and compassion fatigue. There is a significant relationship between these factors, with higher levels of postcode stress contributing to higher levels of compassion fatigue and its subscales: burnout and secondary traumatic stress. These results highlight the importance of addressing and managing the psychological well-being of nurses in resuscitation settings to mitigate the adverse effects of stress and promote their overall resilience and well-being.

## Introduction

Critical care nurses (CCNs) who perform cardiopulmonary resuscitation (CPR) on patients experience high levels of stress, emotional exhaustion, guilt, burden, and anxiety [1]. This stress is associated with an increased risk of developing compassion fatigue symptoms, negatively impacting healthcare professionals' mental health and well-being [2].

Compassion fatigue, burnout, secondary traumatic stress (STS), and vicarious traumatization are interrelated responses to the emotional toll of assisting people in distress [3]. Compassion fatigue involves exhaustion due to prolonged empathy and burnout is a broader workplace-related exhaustion. STS and vicarious traumatization result from indirect exposure to trauma in caregiving, with the latter signifying changes in beliefs and values. These terms collectively address the emotional consequences of providing support to those in need [3].

It’s important to clarify that burnout and secondary traumatic stress, as defined by Stamm (2010), are distinct outcomes that result from exposure to challenging situations [3]. Burnout and secondary traumatic stress are not the same because they emerge from different coping mechanisms that individuals employ when faced with stressors [4]. Burnout typically stems from an assertiveness-goal achievement response. This occurs when an individual struggles to meet personal or professional goals, leading to frustration, loss of control, increased efforts, and decreased morale [4]. In essence, burnout is a response to unmet personal or career aspirations.

On the other hand, secondary traumatic stress arises from a rescue-caretaking response. It occurs when an individual is unable to prevent harm or suffering from someone they are trying to help or care for leading to feelings of guilt and distress [4]. Compassion fatigue is the emotional exhaustion arising from extended, continuous, and close interaction with traumatized patients and their families. It stems from the stress and emotional investment healthcare professionals put into their work, often called the "cost of caring [2].

Compassion fatigue is common among healthcare providers, particularly those working with individuals who have experienced trauma such as natural disasters, accidents, or life-threatening situations. Compassion fatigue is characterized by a depletion of emotional and physical energy, decreased empathy, and a sense of disconnection from patients. CCNs who experience compassion fatigue may feel overwhelmed and emotionally exhausted and may even develop symptoms of anxiety or depression [5].

Incorporating burnout and secondary traumatic stress into the concept of compassion fatigue highlights the interplay between these elements and the complex nature of caregivers' emotional and psychological challenges. Compassion fatigue represents the overarching phenomenon encompassing burnout and secondary traumatic stress, emphasizing the need for comprehensive support and interventions to address the various dimensions of distress experienced by individuals in caregiving professions. Recognizing these interconnected components is crucial for developing effective strategies to manage and mitigate burnout and secondary traumatic stress among healthcare and other caregiving settings [6].

Experiencing stress related to performing CPR can significantly impact compassion fatigue due to the pressure and responsibility of resuscitation efforts, coupled with potential exposure to traumatic outcomes. This chronic stress can deplete nurses' empathetic abilities and result in compassion fatigue, causing feelings of detachment and decreased job satisfaction [7]. Postcode stress CPR refers to the emotional and psychological impact on healthcare providers, especially nurses, after participating in a high-stress CPR event. CPR is a lifesaving procedure, but it can strain the mental and emotional well-being of those involved in resuscitation efforts [8, 9].

Several studies have highlighted the prevalence of post-CPR stress among nurses and its impact on their mental health. Koželj et al., (2021) found that CCNs who participated in a resuscitation attempt reported higher levels of stress and anxiety during the event and increased symptoms of PTSD in the months following the resuscitation attempt [10]. Lee & Cha (2018) reported that performing CPR was a demanding task brought emotional and psychological strain leading to heightened stress. That can be a precursor to compassion fatigue. In addition, nurses facing repeated high-stress CPR may depleted their coping abilities, that can result in emotional exhaustion and reduced empathy [11]. As a result, increased CPR-related stress is likely associated with a higher risk of compassion fatigue. Both Storm & Chen (2021), and Jin et al. (2021) found the nurses who experienced high levels of stress related to their work reported higher levels of burnout and lower levels of job satisfaction [12, 13].

This study is the first to investigate the link between nurses' postcode stress and compassion fatigue in Egypt. It contributes to our understanding of postcode stress and compassion fatigue, conditions both on the rise among healthcare professionals in critical care units. By acknowledging and addressing these issues, healthcare organizations can create a more supportive and sustainable work environment for their employees.

### Aim of the study

- Explore the levels of compassion fatigue and postcode stress among CCNs.
- Assess the relationship between postcode stress and compassion fatigue among CCNs.
- Investigate the effect of postcode stress on compassion fatigue among CCNs.

### Research questions

- What are the levels of compassion fatigue and postcode stress among CCNs?
- Is there a relationship between postcode stress and compassion fatigue among CCNs?
- How does postcode stress affect compassion fatigue among CCNs?

### Materials

#### Study design

A descriptive correlational research design was used.

#### Settings

The study was conducted in five specialized intensive care units (ICUs), each tailored to address distinct categories of critically ill patients. These units comprised a general ICU, trauma ICU, surgical ICU, neurological ICU, and coronary care unit (CCU). The research was carried out in two prominent university hospitals, with these ICUs primarily catering to patients with a wide range of medical conditions and ailments such as trauma, surgical needs, neurological disorders, cardiac conditions.

#### Subjects and sample size

**Fig. 1:**
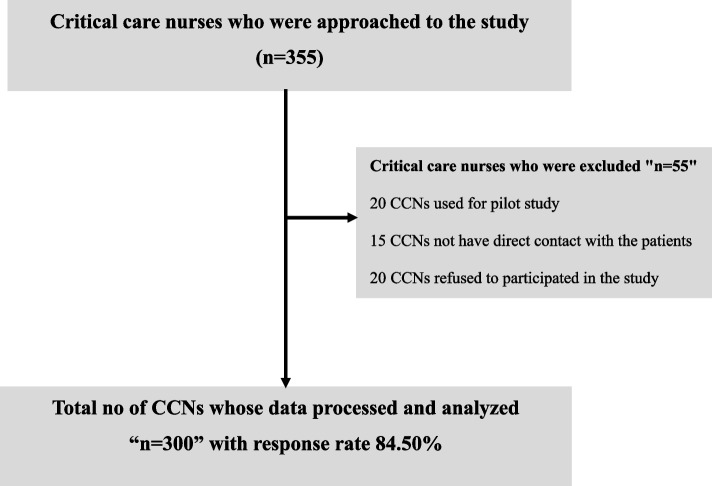
CCNs recruitment process flow chart

Epi info 7 estimated the approached subject size 355 CCNs who working in the two Main University Hospitals, with frequency rate at 50 %, an acceptable error of 5 %, and a confidence coefficient of 99.9% [14]. The inclusion criteria for this study were CCNs with direct patient contact, aged 20 years or older and a valid registered nurse license. A convenience sample of 355 CCNs was initially approached in the study and 55 CCNs were excluded leaving 300. The exclusions were as follows: 20 CCNs were used for the pilot study, 15 CCN nurses did not have direct contact with the patients, and 20 CCN nurses refused to participate. A response rate of 84.50 leaves a total of 300 nurses (See Fig. 1).

## Methods

The subjects were given a clear explanation of the study's objectives and assured that all data would be used solely for research purposes. They were also aware of their right to decline participation or withdraw before finishing the study tools without facing any repercussions. CCNs who participated in the study gave written informed consent. Data was collected over 3 months, from June 2023 to August 2023.

### Study tools that used in data collection

#### Tool I

A questionnaire was containing socio-demographic and work-related characteristics such as age; sex; marital status; level of education; ICU working units and years of experience was used.

#### Tool II: postcode stress scale

It is a self-report questionnaire developed by Cole et al. (2001), to determine nurses' stress during and after resuscitation cases. It included 20 items scored on a 5-point Likert scale ranging from 1 (the claim does not apply to me) to 5 (the claim is typical of me) [15]. The total scores of the scale can range from 20 to 100. Higher scores indicate more significant stress. Scores (20 - 46) indicate a low-stress level; scores (47-73) suggest a moderate stress level, while scores (74 - 100) indicate a high stress level.

Items are grouped into two subscales, the first was: internal source of stress (11 items), which assesses stressors related to conflicting expectations related to their role or behaviors during unsuccessful resuscitation attempts within the individual nurse. The second was, external source of stress (9 items), which assesses stressors between the individual nurse and an external source. Cole and colleagues (2001) reported good internal consistency reliability for this tool, with a Cronbach's alpha coefficient of 0.85 [15]. In the present study Cronbach's alpha coefficient of the postcode stress scale was 0.85.

#### Tool III: Professional Quality of Life Scale (ProQOL)

ProQOL is a 30-item self-report questionnaire developed by Stamm (2010) designed to assess compassion satisfaction, burnout, and secondary traumatic stress experienced by individuals who work in helping professions [3]. In the present study, the researchers used two subscales from this tool of burnout and secondary traumatic stress to measure Compassion Fatigue (CF), and consisted of 10 items each [3].

The scale used a five-point Likert scale, ranging from 1 (never) to 5 (very often). Higher scores on the Burnout and Secondary Traumatic Stress subscales indicate more significant levels of compassion fatigue. Burnout and secondary traumatic stress levels can be categorized into three distinct ranges. Individuals scoring less than 22 fell within the low level, and those with scores between 23 and 41 had a moderate level; and, individuals scoring more than 42 were in the high level. Total scores on the compassion fatigue scale ranged from 20 to 100, with higher scores indicating more feeling of compassion fatigue by the nurses performing CPR. Scores between 20 and 46 indicate a low level; scores between 47 and 73 suggest a moderate level, while scores between 74 and 100 indicate a high level of compassion fatigue. The subscales have been reported to have statistically acceptable internal consistency and reliability values, ranging from 0.75 to 0.88 [16]. In the present study, the subscales have been reported to have statistically acceptable internal consistency and reliability values, ranging from 0.83 to 0.91.

### Validity

The questionnaires were translated into Arabic and then back into English. A panel of five experts, including four professors from psychiatric and mental health nursing and one from the critical care nursing department, evaluated the content and provided feedback on question types, clarity, and overall content validity. Their input was carefully reviewed to ensure accuracy and maintain the study's integrity.

### Pilot study

The researchers conducted a pilot study on 10 % of CCNs (*n* = 20) before implementing the actual study to ascertain the clarity and applicability of the study tools after face validity was done and estimate the time required to complete the study scales. Then, the pilot's findings indicated that no changes were necessary.

### Ethical consecrations

The approval of the Research Ethics Committee (REC), Faculty of Nursing, Alexandria University, was obtained for the study settings to gather the necessary data (IRB00013620-AU-20-5-180-2023). An official letter was also issued from the Faculty of Nursing, Alexandria University, to Alexandria's and Damanhour's Main university hospitals to obtain their permission to collect necessary data.

### Data collection

The approached CCNs in Alexandria's and Damanhour's two governmental university hospitals were met individually by face-to-face appointment in a quiet place during the break time in the hospital and between shift assignments for 20-30 minutes using the three tools to collect data after obtaining their written informed consent.

### Statistical analysis of the data

Data were entered to the computer and analyzed using IBM SPSS software package version 23.0. Quantitative data were described as mean and standard deviation. For normally distributed quantitative variables, one way ANOVA test was used to compare between more than two categories. Student t-test was used to compare two categories for normally distributed quantitative variables. Pearson coefficient was used to correlate between customarily distributed quantitative variables. Simple linear regression analysis was used to determine the effect of postcode stress on compassion fatigue. The significance of the obtained results was judged at the 5% level.

## Results

Table 1 presents data from 300 CCNs that were used for data analysis. The nurses' ages ranged from 20 to over 50 years, with the majority between 20–30 (40.7%). The mean age was 33.10 years, with a standard deviation of 8.57. Regarding gender, the participants mainly comprise females (58%) compared to males (42%). Regarding marital status, most of them were either single (41%) or married (42.3%). A small percentage of the sample was divorced (9.7%) or widowed (7%). In terms of the working department, the majority of the ICU nurses worked in the general ICU (57.3%), followed by the coronary ICU (19.6%), surgical ICU (12.7%), trauma ICU (7.7%), and neurological ICU (2.7%). The level of education was primarily technical (graduate from technical institute of health nursing) (45.3%) or bachelor's degree (graduate from faculty of nursing) (49%), with only a small percentage holding a master's degree (5.7%). Regarding years of experience, the majority of ICU nurses have more than five years of experience (60%), followed by five years of experience (24.7%) and less than five years of experience (15.3%). Finally, the majority of CCNs performing 10–20 CPRs per year (40.7%).

**Table 1:** Distribution of the studied sample according to demographic data (*n*= 300)

**Demographic data**	**No**	**%**
**Age**		
20-<30	122	40.7
30-<40	103	34.3
40-<50	61	20.3
50+	14	4.7
**Mean± SD**	33.10 ± 8.57	
**Gender**		
Male	126	42.0
Female	174	58.0
**Marital status**		
Single	123	41.0
Married	127	42.3
Divorced	29	9.7
Widow	21	7.0
**Working**		
General ICU	172	57.3
Trauma ICU	23	7.7
Surgical ICU	38	12.7
Neurological ICU	8	2.7
Coronary ICU	59	19.6
**Level of education**		
Technical	136	45.3
Bachelor	147	49.0
Master	17	5.7
**Years of experience**		
<5 years	46	15.3
5years	74	24.7
>5 years	180	60.0
**Number of CPR per years**		
1-10	106	35.3
10-20	56	18.7
more than 15	138	46

Tables 2 and 4 present the results of the Postcode Stress Scale, which measures internal and external sources of postcode stress experienced by CCNs during code situations. The scale includes eleven items measuring internal and nine measuring external sources of postcode stress. The mean score for internal sources of postcode stress that measured stress results from conflicting expectations within the individual nurse, such as “my hands shake during a code and I feel like I didn’t function well during a code,” was 35.50, with a standard deviation of 9.21 and a mean percent score of 55.69%. This suggests that the CCNs in the study experienced moderate postcode stress levels due to internal factors during code situations. The mean score for external sources of postcode stress that measured conflicting expectations between the nurses and another source, such as “my peers are quick to notice and point out that I made a mistake and a nurse manager/supervisor criticizes me when I’ve done my best during a code situation,” was 32.48, with a standard deviation of 8.46 and a mean percent score of 65.21%. This indicates that the CCNs in the study experienced high postcode stress levels due to external factors during code situations. The total score for overall postcode stress is 67.98, with a standard deviation of 16.39 and a mean percent score of 59.98%. This suggests CCNs experienced moderate postcode stress levels during code situations. Overall, the results indicate that both internal and external sources of postcode stress contribute significantly to the stress experienced by CCNs during CPR code situations.

**Table 2:** Description of mean and standard deviation of Post-Code Stress Scale

**Post-Code Stress Scale**	**Mean**	**SD**
**Internal sources of stress**		
When my hands shake during a code	3.50	1.20
When I feel like I didn’t function well during a code	3.29	1.14
When I lose my confidence during a code	3.58	1.18
When I am unable to make a properly functioning piece of equipment operate during a code	2.88	1.10
When we code a patient, I believe we should not code	3.91	1.06
When I have trouble reading the ECG	3.26	1.16
When I wonder if I made a mistake	3.30	1.27
When I am not permitted time to regroup and pick myself up after a code	3.36	1.40
When I code someone young	2.74	1.28
When I think I might have missed a sign or symptom that would have helped me predict that the patient would code	2.84	1.30
When the patient dies	2.83	1.18
**Total score Internal sources of stress**	**35.50**	**9.21**
**External sources of stress**		
When my peers are quick to notice and point out that I made a mistake	3.55	1.20
When more than one doctor gives orders during a code	3.41	1.07
When a patient’s family thinks I can keep him/her alive	3.67	1.04
When hospital policies/procedures are conflicting	3.85	1.20
When I code some patients only because hospital policy says I must	3.98	1.31
When a nurse manager/supervisor criticizes me when I’ve done my best	3.47	1.21
When a nurse manager doesn’t provide assistance during a code	3.54	1.25
When people think I can function appropriately immediately after a code	3.50	1.10
When no one talks about the code after it is over	3.50	1.32
**Total score External sources of stress**	**32.48**	**8.46**
**Total score overall stress**	**67.98**	**16.39**

Post-Code Stress Scale

20 – 46 low

47 – 73 moderate

74 – 100 high

Tables 3 and 4 present the results of Compassion Fatigue and its subscales, which measure burnout and secondary traumatic stress among CCNs. The subscales include 20 items, with 10 items measuring burnout and the other 10 measuring secondary traumatic stress. The mean score for the burnout subscale is 32.60, with a standard deviation of 8.91 and a mean percent score of 56.51%. This indicates that the study's CCNs experienced moderate burnout. As well as the mean score for the secondary traumatic stress subscale is 32.80, with a standard deviation of 6.41 and a mean percent score of 57.0%. This suggests that CCNs experienced moderate to high levels of secondary traumatic stress. The data showed that the healthcare professionals in the sample experienced a moderate level of compassion fatigue.

**Table 3:** Description of mean and standard deviation of Compassion Fatigue and its subscales

**Compassion Fatigue Scale (CFS)**	**Mean**	**SD**
**Burnout Subscale**		
I am happy^a^	3.20	1.16
I feel connected to others.^a^	3.10	1.22
I am not as productive at work because I am losing sleep over traumatic experiences of a person I [help].	3.21	1.08
I feel trapped by my job as a [helper].	3.35	1.24
I have beliefs that sustain me.^a^	3.18	1.14
I am the person I always wanted to be.^a^	3.18	1.04
I feel worn out because of my work as a [helper].	3.28	1.12
I feel overwhelmed because my case [work] load seems endless.	3.50	1.06
I feel "bogged down" by the system.	3.06	1.19
I am a very caring person^a^	3.55	0.99
**Total score Burnout Subscale**	**32.60**	**8.91**
**Secondary Traumatic Stress Subscale**		
I am preoccupied with more than one person I [help]	3.08	1.13
I jump or am startled by unexpected sounds	3.27	1.01
I find it difficult to separate my personal life from my life as a [helper].	3.12	1.16
I think that I might have been affected by the traumatic stress of those I [help].	3.49	1.10
Because of my [helping], I have felt "on edge" about various things.	3.34	1.19
I feel depressed because of the traumatic experiences of the people I [help].	3.53	1.10
I feel as though I am experiencing the trauma of someone I have [helped].	3.08	1.13
I avoid certain activities or situations because they remind me of frightening experiences of the people I [help]	3.27	1.01
As a result of my [helping], I have intrusive, frightening thoughts	3.12	1.16
I can't recall important parts of my work with trauma victims.	3.49	1.10
**Total score Traumatic Stress Subscale**	**32.80**	**6.41**
**Total score Compassion Fatigue Scale (CFS)**	**65.40**	**14.34**

^a^reversed score items

Burnout and secondary-Traumatic Stress Subscales:

10 - 22 low.

23 - 35 moderate

36- 50 high

The Compassion Fatigue Scale (CFS):

20 – 46 low

47 – 73 moderate

74 – 100 high

**Table 4:** Distribution of the levels and frequency of compassion fatigue and post code stress

**Levels**	**Low**		**Moderate**		**High**	
	**No.**	**%**	**No.**	**%**	**No.**	**%**
**Compassion Fatigue Subscales**						
Burnout Subscale	29	9.7	213	71.0	58	19.3
Secondary Traumatic Stress Subscale	3	1.0	267	89.0	30	10.0
**Compassion Fatigue Scale**	13	4.3	184	61.3	103	34.4
**Post-Code Stress Scale Subscales**						
Internal sources of stress	27	9.0	183	61.0	90	30.0
External sources of stress	42	14.0	116	38.7	142	47.3
**Post-Code Stress Scale**	37	12.3	152	50.7	111	37.0

Table 5 presents the correlation coefficients between postcode stress and compassion fatigue with its subscales. The correlation coefficients measure the strength and direction of the relationship between the different variables. The results show that there is a statistically significant positive correlation between the burnout subscale and internal sources of postcode stress (*r* = 0.372, *p* < 0.001), external sources of postcode stress (*r* = 0.274, *p* < 0.001), and overall postcode stress (*r* = 0.350, *p* < 0.001). This indicates that as the postcode stress experienced by CCNs during code situations increased, burnout also increased. Finally, there is a statistically significant positive correlation between secondary traumatic stress and internal sources of postcode stress (*r* = 0.469, *p* < 0.001), external sources of postcode stress (*r* = 0.494, *p* < 0.001), and overall postcode stress (*r* = 0.518, *p* < 0.001). This suggests that as the postcode stress experienced by CCNs during code situations increased, secondary traumatic stress also increased. There is a statistically significant positive correlation between postcode stress and compassion fatigue (*r*=0.449, *p*=0.001). In other words, higher stress levels related to postcode experiences are associated with a higher likelihood of experiencing compassion fatigue among nurses.

**Table 5:** Correlation between Post-Code Stress Scale and Compassion Fatigue with its subscales

**Study variables**		**Burnout**	**Secondary-Traumatic Stress**	**Compassion fatigue**
**Internal sources of stress**	**r**	0.372^*^	0.469^*^	0.441^*^
**External sources of stress**	**r**	0.274^*^	0.494^*^	0.391^*^
**Post code stress**	**r**	0.350^*^	0.518^*^	0.449^*^

r: Pearson coefficient

^*^: Statistically significant at *p* ≤ 0.001

In Table 6, simple linear regression analyses were conducted to understand how the postcode stress predicts compassion fatigue" and its various subscales, including burnout" and traumatic stress. For the burnout subscale, the analysis revealed that postcode stress has a statistically significant positive impact on burnout." Specifically, for every unit increase in postcode stress, there is a corresponding increase of 0.381 units in burnout. This relationship is statistically significant, as indicated by the high t-value (6.460) and *p*-value (<0.001), suggesting that the association is not due to chance. The confidence interval (CI) of 95% indicates that the effect of postcode stress on burnout falls within the range of 0.265 to 0.497. The model's adjusted (R2) of 0.120 suggests that postcode stress explains approximately 12 % of the variance in burnout. For the secondary traumatic stress subscale, the analysis also demonstrated that postcode stress significantly influences secondary traumatic stress. For every unit increase in postcode stress, there is a corresponding increase of 0.405 units in secondary traumatic stress. This relationship is highly statistically significant, with a substantial t-value (10.467) and *p*-value (<0.001), further emphasizing its significance. The 95% confidence interval (CI) ranges from 0.329 to 0.482. The model's adjusted (R2) of 0.266 indicates that postcode stress accounts for approximately 26.6% of the variance in secondary traumatic stress.

**Table 6:** Simple linear Regression Analyses for Post-Code Stress Scale predicting Compassion Fatigue and its subscales

**Compassion Fatigue subscales / Burnout**						
	B	Beta	t	*p*	95% CI	
					**LL**	**UL**
**Post-Code Stress**	0.381	0.350	6.460^*^	<0.001^*^	0.265	0.497
*R*^2^=0.123 ,Adjusted *R*^2^ = 0.120, F= 41.731^*^, *p*<0.001^*^						
**Compassion Fatigue subscale / Secondary Traumatic Stress**						
**Post-Code Stress**	0.405	0.518	10.467^*^	<0.001^*^	0.329	0.482
*R*^2^= 0.269, Adjusted *R*^2^=0.266, F= 109.556^*^, *p*<0.001^*^						
**Total Compassion Fatigue**						
**Post-Code Stress**	0.462	0.449	8.668^*^	<0.001^*^	0.357	0.567
*R*^2^= 0.201, Adjusted *R*^2^=0.199, F= 75.136^*^, *p*<0.001^*^						

F,p: f and *p* values for the model

R^2^: Coefficient of determination

B: Unstandardized Coefficients

Beta: Standardized Coefficients

t: t-test of significance

LL: Lower limit UL: Upper Limit

^*^: Statistically significant at *p* ≤ 0.05

Regarding total compassion fatigue, the analysis also reveals that postcode stress has a statistically significant positive effect on total compassion fatigue. A unit increase in postcode stress corresponds to a 0.462-unit increase in compassion fatigue. This relationship is highly statistically significant, as evidenced by the substantial t-value (8.668) and *p*-value (<0.001). The 95% confidence interval (CI) spans from 0.357 to 0.567. The model's adjusted (R2) of 0.199 suggests that postcode stress explains approximately 19.9% of the variance in total compassion fatigue.

## Discussion

Burnout and compassion fatigue can impact critical and emergency care nurses during CPR situations [17]. To prevent burnout and maintain their ability to provide high-quality patient care during CPR, healthcare professionals must assess the compassion fatigue and stress that arise in challenging situations like CPR [17]. CCNs, as part of a multidisciplinary team, must have basic and advanced resuscitation skills, which exposes them to heavy stress, such as internal conflicts within a nurse, such as feeling uncertain about one's competence despite their knowledge about CPR, or external conflicts between the nurse and other sources, such as when more than one doctor gives orders during CPR situations [10]. This is the first Egyptian study to explore the relationship between stress related to CPR and compassion fatigue from CCNs experiences.

Regarding postcode stress levels, the studied CCNs had moderate to high postcode stress levels caused by source of stress may arise from conflict within the individual nurse or between the individual nurse and another source like a hospital policy or the nurse administrator that happened during code situations. Factors that increase postcode stress among CCNs might include the emotional intensity of the resuscitation procedure, which can be painful and emotionally hard for healthcare personnel [18]. Nurses who watch patients' suffering and death during resuscitation may feel guilty, powerless, and anxious. Furthermore, the physical difficulties of CPR might lead to postcode stress among nurses. CPR providers may suffer from physical tiredness, muscular strain, and injury, which can worsen feelings of stress and burnout [18]. This is supported by McMeekin et al. (2017), who surveyed 490 CCNs and reported that CCNs exhibit moderate levels of psychological stress and PTSD symptoms [1]. When asked to recollect failure resuscitation and the coping mechanisms utilized, Meyer and colleagues (2015) reported that high stress levels in nurses working in ICU units increase their CF and affect job satisfaction [19]. In the Middle East and North Africa region, the prevalence range of low, moderate, and high perceived stress among nurses was 0.8–65%, 5.9–84.5%, and 6.7–99.2%, respectively [20]. In the United States, a study found that critical care nurses show moderate levels of postcode stress and PTSD symptoms when asked to recall an unsuccessful resuscitation [21].

The current studied nurses experienced moderate to high levels of burnout, secondary traumatic stress, and subsequently moderate to high levels of compassion fatigue. Compassion fatigue is typical among nurses who work in intensive care units and offer high-stress care to patients [22]. There are several factors that impact CF in ICU setting. One factor is extended exposure to stressful situations, such as watching patients' suffering and death. CCNs may also face a heavy workload, lengthy shifts, and a lack of support from colleagues, all of which can lead to emotional weariness and burnout. The high workload significantly predicted compassion fatigue among CCNs [22]. In addition, a lack of support from colleagues was linked to increased compassion fatigue among CCN healthcare staff [23]. In a multinational survey of 159 ICUs in 16 Asian countries, both physicians and nurses had high levels of burnout (50.3% versus 52.0%). Among countries or regions, burnout rates ranged from 34.6 to 61.5%1 [24]. As well a systematic review found that burnout is highly prevalent among healthcare providers across countries in the Middle East [25].

Consequently, experiences of sudden death or significant loss can lead to the accumulation of negative emotions. Over time, these emotions can contribute to the emergence of CF. As nurses experience these negative emotions, they may feel helpless to prevent patients' health from declining further, leading to heightened death anxiety [26, 27].

Compared to Emergency nurses are at risk of CF due to the stresses of caring for patients who are in significant emotional pain and physical distress. Approximately 82% of emergency nurses had moderate to high levels of burnout, and nearly 86% had moderate to high levels of compassion fatigue [28]. Moreover, In Li et al., 2022 focus was on Compassion fatigue and compassion satisfaction among Chinese palliative care nurses. The research revealed that levels of compassion fatigue, encompassing burnout and secondary traumatic stress, were elevated compared to other nursing specialties. The study identified various factors influencing compassion fatigue and compassion satisfaction, including individual, work-related, and psychosocial aspects [29].

The current study’s findings reveals a positive correlation between postcode stress and burnout, suggesting that the more CCNs experienced postcode stress during code situations, the more they suffer from burnout. Postcode Stress explains approximately 12 % of the variance in "Burnout" among CCNs, highlighting its importance as a predictor of this aspect of compassion fatigue. CPR is a high-stress emergency requiring providers to do quick and effective chest compressions, administer drugs, and manage airway and breathing. Stress can impair cognitive and motor abilities, making it harder for providers to administer CPR efficiently, resulting in tiredness. According to Vincent et al. (2021), healthcare personnel who reported higher stress levels had a worse capacity to deliver efficient CPR [18]. Also, CPR is one of the nurse's occupational stressors that may hurt their mental health and lead to burnout, fatigue, and symptoms of post-traumatic stress, which affect their professionalism, quality of life, increase absenteeism, and loss of productivity [30].

Also, the current study indicates a significant positive correlation between postcode stress and secondary traumatic stress. As postcode stress increases, so does the level of secondary traumatic stress experienced by the participants. The coefficient of determination values demonstrated a statistically significant and moderately to strongly positive relationship between postcode stress and secondary traumatic stress. Postcode stress explains approximately 26.6% of the variance in secondary traumatic stress among the participants, highlighting its significance as a predictor of this aspect of compassion fatigue. The results also suggested a significant positive relationship between postcode stress and compassion fatigue among CCNs. The Beta coefficient indicates that higher levels of postcode stress were associated with higher levels of compassion fatigue. As well as postcode stress explains 19.9% of the variance in compassion fatigue. This means other factors not included in the model may also contribute to developing compassion fatigue among CCNs.

Similarly, Ali et al. (2022) found a significant predictive relationship between stress and Compassion Fatigue (CF) in emergency nurses trained in CPR [31]. Similarly, Mealer et al. (2016) observed that healthcare personnel experiencing higher stress levels were more prone to developing post-traumatic stress symptoms following a cardiac arrest incident [32]. This association could be attributed to the considerable emotional stress experienced by healthcare providers when dealing with patients' encounters with death, dying, and resuscitation efforts [33].

### Weakness of the study

The study did not investigate other factors affecting compassion fatigue among CCNs, such as social support, coping strategies, or personality traits. The study used a convenience sampling method, which may introduce bias into the sample composition [34]. The data collected in this study relied on self-report measures subject to social desirability bias [34]. Participants may provide responses they believe are expected or acceptable rather than their true feelings or experiences. The findings of this study may have limited generalizability to CCNs in other regions or healthcare systems, and caution should be exercised when applying the results to broader populations. Further research is needed to better understand the nuances of CF and postcode stress in different cultural and healthcare contexts.

## Conclusion

In conclusion, caring for critically ill patients and facing the challenges in ICU can contribute to the development of postcode stress and compassion fatigue among CCNs. The study identified a significant positive correlation between postcode stress and compassion fatigue, particularly secondary traumatic stress. This suggests that the stress associated with CPR and resuscitation efforts predicts the development of compassion fatigue symptoms among CCNs. By incorporating burnout and secondary traumatic stress into the concept of compassion fatigue, this study highlights the interplay between these elements and provides a more comprehensive understanding of healthcare professionals' emotional and psychological challenges.

### Implication in nursing practice

Healthcare organizations need to take into account the specific and often overwhelming stress that CCNs undergo when participating in CPR and resuscitation procedures.

They should provide adequate resources, including mental health support, debriefing sessions, and access to counseling services, to help nurses cope with the emotional toll of their work. Hospitals and educational institutions should offer training and education programs that equip CCNs with effective coping strategies and stress management techniques. This can help them better navigate the challenges of their profession. Moreover, nursing implications involve internal and external strategies to mitigate stress related to conflicting expectations in resuscitation situations. These efforts should focus on enhancing CCNs' confidence, addressing institutional policies, promoting open communication, and fostering a supportive work culture to ensure optimal patient care and nurse well-being.

## Data Availability

Data will be available on reasonable request from the corresponding author.
